# Pembrolizumab-Induced Isolated Cranial Neuropathy: A Rare Case Report and Review of Literature

**DOI:** 10.3389/fneur.2021.669493

**Published:** 2021-05-11

**Authors:** Francesco Bruno, Rosa Antonietta Palmiero, Bruno Ferrero, Federica Franchino, Alessia Pellerino, Enrica Milanesi, Riccardo Soffietti, Roberta Rudà

**Affiliations:** ^1^Department of Neuro Oncology, University Hospital of the City of Health and Science of Turin, Turin, Italy; ^2^Department of Neurology, University Hospital of the City of Health and Science of Turin, Turin, Italy; ^3^Department of Oncology, University Hospital of the City of Health and Science of Turin, Turin, Italy; ^4^Department of Neurology, Castelfranco Veneto Hospital, Castelfranco Veneto, Italy

**Keywords:** pembrolizumab, anti-PD1 agents, neurological immune-related adverse effects, immune-related neurological complications, autoimmune neuropathy, case report

## Abstract

**Introduction:** Anti-PD1 agents are widely used in the treatment of solid tumors. This has prompted the recognition of a class of immune-related adverse events (irAEs), due to the activation of autoimmune T-cells. Pembrolizumab is an anti-PD1 agent, which has been related to an increased risk of various neurological irAE (n-irAEs). Here, we present a rare case of pembrolizumab-induced neuropathy of cranial nerves.

**Case Report:** A 72-year-old patient was diagnosed with a lung adenocarcinoma in February 2018 (EGFR–, ALK–, and PDL1 90%). According to the molecular profile, pembrolizumab was started. After three administrations, the patient developed facial paresis, ptosis, ophthalmoplegia, and dysphonia. As brain metastases and paraneoplastic markers were excluded, a drug-related disorder was suspected and pembrolizumab was discontinued. A nerve conduction study and electromyography excluded signs of neuropathy and myopathy at four limbs, and repetitive nerve stimulation was negative. However, altered blink reflex and nerve facial conduction were consistent with an acute neuropathy of the cranial district. Thus, the patient was treated with two cycles of intravenous immunoglobulins (IVIg), which rapidly allowed improvement of both symptoms and neurophysiological parameters. However, the patient died in October 2018 for a progression of lung tumor.

**Discussion:** Only 16 cases of pembrolizumab-related neuropathies have been described so far. Our case is of particular interest for the isolated involvement of cranial nerves and the prompt response to IVIg.

**Conclusion:** N-irAEs are insidious conditions that require solid knowledge of onco-immunotherapy complications: it is mandatory not to delay any treatment that would potentially modify the course of a neurological complication.

## Introduction

Pembrolizumab (an anti-PD1 agent) may favorably impact the outcome of melanoma and non-small cell lung carcinoma (NSMLC) (1, 2). By promoting the activation of T-cells, pembrolizumab fosters the immune response against tumor. However, it may also increase the risk of autoimmune reactions, known as immune-related adverse events (irAEs). Various neurological irAEs (n-irAEs) have been associated with pembrolizumab: in clinical trials with checkpoint inhibitors, 6.3% of patients on pembrolizumab presented n-irAEs of any type and grade (3). The peripheral nervous system is more likely to be involved than the central nervous system (4). In a recent systematic review focused on pembrolizumab-induced neuromuscular disorders (5), 14 (36%), 13 (33%), 9 (23%), and 3 (8%) of 39 patients on pembrolizumab were reported to develop myopathy, myasthenia gravis, neuropathy, or overlapping disorders, respectively.

Here, we describe a patient who developed a rare acute neuropathy of cranial nerves from pembrolizumab.

## Case Report

In February 2018, a 72-year-old man was diagnosed with an adenocarcinoma of the lung (EGFR–, ALK–, and PDL1 amplificated in 90% of the cells). A total-body CT scan and an FDG-PET ruled out the presence of metastases at presentation. Based on the molecular profile, pembrolizumab was started. After three cycles (June 2018), the patient developed fatigue, dizziness, mild bilateral facial palsy (grade III of the House–Brackmann scale), bilateral ptosis and ophthalmoplegia, dysphonia, and dysphagia. As the brain and spine MRI with gadolinium excluded the occurrence of metastases, a neuroimmunological drug-related disorder or a paraneoplastic syndrome was considered, and pembrolizumab was stopped. First, we ruled out the presence of neuromuscular junction disorders: both repetitive nerve stimulation (RNS) and specific antibody assays—including anti-acetylcholine receptor (AChR), anti-muscle-specific kinase (MuSK), and P/Q-type VGCC antibodies—were negative. Second, we tested the markers of immune-mediated neuropathy (anti-MAG, anti-GM1/2, anti-GD1a/b, and anti-GQ1b antibodies) and paraneoplastic syndromes (anti-Tr, anti-CV2/CRMP5, anti-amphiphysin, anti-PNMA2/TaMa, anti-GAD65, anti-recoverin, anti-Ri, anti-Yo, anti-Hu, anti-Zic4, anti-SOX1, and anti-titin antibodies), with negative results. Also, creatine kinase was normal (80 IU/l), and cerebrospinal fluid (CSF) did not harbor any inflammatory alterations (being cell count 5/mm^3^ and protein concentration 0.32 g/l). Then, we performed nerve conduction studies (NCS) and electromyography (EMG) at the limbs and cranial district: while no signs of neuropathy or myopathy were seen at the extremities, the evidence of slightly decreased amplitude of facial nerve conduction and altered blink reflex (lacking both ipsilateral and contralateral R2 components) suggested a diagnosis of a neuropathy involving the cranial nerves (Tables 1A,B). Therefore, in July 2018, the patient was treated with intravenous immunoglobulins (IVIg: 0.4 g/kg/5 days), with no use of oral glucocorticoids due to the presence of moderate dysphagia. The therapy was well-tolerated and allowed a prompt relief from dizziness, diplopia, and dysphonia and total remission of facial palsy. Also, NCS of the facial nerves and blink reflex showed a rapid improvement, as both ipsilateral and contralateral R2 components were almost completely restored after the first cycle of IVIg (Tables 1A,B). Due to the rapid improvement of symptoms, the employment of intravenous steroids was not needed, but a second cycle of IVIg was administered in August 2018 to consolidate the result. The neurological condition remained stable until October 2018, when the patient died for a progression of the primary tumor.

**Table 1A:** Nerve conduction study of the facial nerves at presentation, after the first cycle of IVIg, and after the second cycle.

**Nerve**	**At presentation**		**After 1st cycle of IVIg**		**After 2nd cycle of IVIg**	
	**Latency onset**	**Amplitude**	**Latency onset**	**Amplitude**	**Latency onset**	**Amplitude**
	**ms**	**mV**	**Ms**	**mV**	**ms**	**mV**
**Left n. facialis**						
Mandible—orbicularis oculi	2.26	2.1	3.2	2.8	2.5	3.4
Mandible—nasalis	3.04	1.57	2.96	2.2	2.11	2.5
**Right n. facialis**						
Mandible—orbicularis oculi	2.65	1.79	3.28	3.3	2.81	3.3
Mandible—nasalis	3.82	2.1	3.04	2.3	3.74	2.3

*Compound muscle action potential (CMAP) amplitude increased to normal values since after the first cycle of therapy*.

**Table 1B:** Latencies of R1 and R2 components of blink reflex at presentation, after the first cycle of treatment, and after the second cycle.

**Stimulation**	**Registration**	**At presentation**				**After 1st cycle of IVIg**				**After 2nd cycle IVIg**			
		**R1-latency**		**R2-latency**		**R1-latency**		**R2-latency**		**R1-latency**		**R2-latency**	
		**ms**	**RefDev**	**ms**	**RefDev**	**ms**	**RefDev**	**Ms**	**RefDev**	**ms**	**RefDev**	**ms**	**RefDev**
Left	Left	12.0	1.85	A	NA	11.5	1.24	45.7	4.5	12.1	1.93	44.6	4.1
	Right			A	NA			48.6	5.3			43.6	3.9
	Difference			NA	NA			−2.9	−2.7			0.96	3.9
Right	Right	11.8	1.6	A	NA	11.8	1.60	39.6	2.7	11.1	0.75	38.1	2.2
	Left			A	NA			44.9	4.2			37.0	1.92
	Difference			NA	NA			−5.2	−4			1.07	−0.31

*Reference values for NCS n. facialis: at orbicularis oculi, latency ≤ 3.1 ms, amplitude ≥1.0 mV; at nasalis, latency ≤ 4.2 ms, amplitude ≥1.0 mV. Reference values for blink reflex: R1 (ipsilateral), latency ≤ 13 ms, difference ≤ 1.2 ms; R2 (ipsilateral), latency ≤ 41 ms, difference ≤ 5 ms; R2 (contralateral), latency ≤ 44 ms, difference ≤ 7 ms.*

*A, absent; NA, not applicable; RefDev, deviation from reference*.

We identified 24 cases of pembrolizumab-induced neuropathies and/or radiculopathies, mostly reported in small series of single or few patients (Table 2) (6–18). Melanoma was the primary tumor in 20 cases, whereas only three patients had lung adenocarcinoma (6, 7, 16). Fourteen patients were treated with pembrolizumab as a single agent (three of them had been previously treated with ipilimumab), three with a combination of pembrolizumab with chemotherapy, and seven with an association of pembrolizumab and ipilimumab. Seven patients developed acute demyelinating polyradiculopathy involving the lower and upper extremities, thus mimicking Guillain-Barré syndrome (GBS); three presented involvement of both limbs and cranial district, similar to GBS—Miller Fisher variant; and only five isolated neuropathies of cranial nerves were described (8, 18). Immune-mediated neuropathy occurred with a median latency of four cycles. The diagnostic workup included nerve conduction studies (NCS) and electromyography (EMG) in 18 patients, lumbar puncture in 14 patients, and dosage of serum antibodies of autoimmune neuropathies or paraneoplastic syndromes in eight cases. CSF harbored albuminocytologic dissociation in five cases (7, 10, 11, 14), while in seven cases, it showed pleocytosis (7, 10, 12, 13, 17, 18), and in two cases, it was normal (18).

**Table 2:** Pembrolizumab-induced neuropathy: review of literature.

**Author**	**Patients**	**Cancer** **diagnosis**	**Treatment**	**Cycles to** **onset of** **n-irAE**	**Neurological presentation**		**Diagnostic workup**					**Diagnosis**	**Pembrolizumab** **stopped**	**Management of** **the n-irAE**	**Outcome** **(of the** **n-irAE)**
					**Limb** **weakness** **and/or** **sensory** **disorder**	**Cranial nerve** **involvement**	**CSF**		**NCS/EMG/** **evoked** **potentials**	**Autoimmune** **antibodies**	**Exclusion of** **paraneoplastic** **syndrome**				
Aya et al. (6)	1	Melanoma	Pembrolizumab (previous treatments: IFN-alpha, dacarbazine, and ipilimumab)	1	Yes	Yes (palsy of the abducens nerve)	NA		Sensory peripheral polyneuropathy	NA	NA	Vasculitic neuropathy (confirmed by nerve and muscle biopsy)	Yes	Oral and intravenous glucocorticoids	Improved
de Maleissye et al. (7)	2	Melanoma	Pembrolizumab	2	Yes	Yes (facial palsy)	Pleocytosis (45 cells/mm^3^), slight increase of proteins (0.56 g/l)	No A-C dissociation	Demyelinating polyradiculopathy	NA	Yes	GBS, Miller-Fisher variant	Yes	IVIg	Improved
	3	Melanoma	Ipilimumab + pembrolizumab	6	Yes	No	Normal cells count; slight increase of proteins (0.74 g/l)	A-C dissociation	Demyelinating polyradiculopathy	NA	Yes	CIDP	Yes	Oral and intravenous glucocorticoids + PEX	Not improved
Zimmer et al. (8)	4	Melanoma	Pembrolizumab (previous treatments: IFN-alpha, dacarbazine, and ipilimumab)	4	NA	Yes (paresis of the oculomotor nerve)	NA		NA	NA	NA	Neuritis of the oculomotor nerve	Yes	Prednisolone	Improved
	5	Melanoma	Pembrolizumab (previous treatments: IL2, dabrafenib/trametinib and ipilimumab)	11	Yes	No	NA		NA	NA	NA	GBS	Yes	Prednisolone	Improved
Diamantopoulos et al. (9)	6	Melanoma	Pembrolizumab	1	Yes	No	NA		Axonal polyneuropathy and myositis	Ab anti-neuronal antigens - Ab anti-gangliosides - Ab related to myositis -	Yes	Overlapping axonal polyneuropathy and myositis	Yes	Methylprednisolone + IVIg + PEX	Deceased
Kao et al. (10)	7	Melanoma	Pembrolizumab	10	Yes	No	Normal cell count (2 cells/ mm^3^); slight increase of proteins (0.71 g/l)	A-C dissociation	Demyelinating polyradiculopathy	Ab anti-GM1 - Ab anti-GD1b -	Yes	GBS	Yes	Prednisone + IVIg	Improved
	8	Melanoma	Pembrolizumab	6	Yes	No	NA		Mixed axonal and demyelinating polyneuropathy	NA	NA	Peripheral mixed demyelinating and axonal neuropathy	Yes	Prednisone	Improved
	9	Melanoma	Pembrolizumab	20	Yes	Yes (facial palsy, dysphonia)	Pleocytosis (12 cells/mm^3^); slight increase of proteins (0.95 g/l)	No A-C dissociation	Demyelinating polyradiculopathy	Ab anti-GM1/2 - Ab anti-GD1a/b - Ab anti-GQ1b -	Yes	GBS, Miller-Fisher variant	Yes	IVIg	Improved
Sepúlveda et al. (11)	10	Melanoma	Ipilimumab + pembrolizumab	23	Yes	No	No cells; slight increase of proteins (0.67 g/l)	A-C dissociation	Axonal polyradiculopathy	Ab anti-neuronal antigens - Ab anti-gangliosides -	Yes	GBS, AMAN variant	Yes	IVIg + PEX	Improved
Yost et al. (12)	11	Melanoma	Ipilimumab + pembrolizumab	3 months after pembrolizumab dismissal^‡^	No	Yes (facial palsy, dysphonia)	Pleocytosis (12 cells/mm^3^); high proteins level (1.95 g/l)	No A-C dissociation	Altered blink reflex (absent R1/R2 responses)	Ab anti-GM1/2 - Ab anti-GD1a/b - Ab anti-GQ1b -	Yes	Isolate acute neuropathy of facial nerve	Yes	Methylprednisolone + IVIg	Improved
Fellner et al. (13)	12	Melanoma	Pembrolizumab	18 weeks after first pembrolizumab administration^‡^	Yes	No	Pleocytosis (58 cells/mm^3^); high proteins level (2.27 g/l)	No A-C dissociation	Demyelinating polyradiculopathy	Ab anti-GD1b - Ab anti-GQ1b - Ab anti-MAG Ab anti-neuronal antigens -	Yes	GBS	Yes	Methylprednisolone	Improved
Manam et al. (14)	13	Lung adenocarcinoma	Pembrolizumab + carboplatin and pemetrexel	2	Yes	No	Slight increase of proteins (0.68 g/l); no cell count reported.	A-C dissociation (as reported by authors)	NA	NA	Yes	GBS	Yes	Methylprednisolone + IVIg + PEX	Improved
	14	Melanoma	Pembrolizumab + dabrafenib and trametinib	2	Yes	No	Slight increase of proteins (0.56 g/l); no cell count reported.	A-C dissociation (as reported by authors)	Demyelinating polyradiculopathy	Ab anti-GM1 -	Yes	GBS	Yes	PEX	Deceased (due to the n-irAE)
Ong et al. (15)	15	Lung adenocarcinoma	Pembrolizumab	2	Yes	Yes (facial palsy)	NA		Demyelinating polyradiculopathy	NA	Yes	GBS, Miller-Fisher variant	Yes	Methylprednisolone + IVIg	Improved
Dubey et al. (16)^§^	16	NA	Ipilimumab + pembrolizumab	1	NA	Yes (bilateral facial palsy)	NA		NA	NA	NA	Bilateral acute neuropathy of facial nerves	NA	NA	NA
	17	Melanoma	Pembrolizumab	2	Yes	No	NA		Lumbosacral radiculopathy and peripheral sensory neuropathy	NA	NA	GBS	Yes	None	Improved
	18	Melanoma	Pembrolizumab	1	Yes	No	NA		Length-dependent sensory and motor axonal polyneuropathy	NA	NA	Acute sensory and motor axonal polyneuropathy	No	Gabapentin 100 mg twice a day	Improved
	19	Lung adenocarcinoma	Erlotinib + pembrolizumab	1	Yes	No	NA		Multiple proximal mononeuropathy of left upper arm	NA	NA	Neuralgic amyotrophy	Yes	Prednisone 60 mg daily	Improved
Muralikrishnan et al. (17)	20	Melanoma	Pembrolizumab	2	Yes	No	Pleocytosis (17 cells/mm^3^); slight increase of proteins (0.78 g/l)	No A-C dissociation	Demyelinating polyradiculopathy	Ab anti-gangliosides - Ab anti-MAG -	NA	GBS	Yes	Methylprednisolone + IVIg + PEX	Improved
Vogrig et al. (18)	21	Melanoma	Pembrolizumab	1	No	Yes (visual loss)	Pleocytosis (34 cells/mm^3^), normal protein content	No A-C dissociation	NA	NA	NA	Optic neuropathy	Yes	None	Improved
	22	Melanoma	Ipilimumab + pembrolizumab	6 months after pembrolizumab initiation^‡^	No	Yes (visual loss)	Normal	No A-C dissociation	Altered visual evoked potentials (VEPs)	NA	NA	Optic neuropathy	Yes	Methylprednisolone	Not improved
	23	Melanoma	Ipilimumab + pembrolizumab	NA	No	Yes (visual / hearing loss)	Normal	No A-C dissociation	Altered visual evoked potentials (VEPs)	NA	NA	Optic neuropathy / auditory neuropathy	Yes	Methylprednisolone + PEX	Not improved
	24	Melanoma	Ipilimumab + pembrolizumab	1 month after pembrolizumab initiation^‡^	No	Yes (palsy of the abducens nerve)	Mild pleocytosis (6 cells/mm^3^), normal protein content	No A-C dissociation	NA	NA	NA	Abducens nerve neuropathy	Yes	Oral glucocorticoids	Improved

*The authors reported “13 weeks after first pembrolizumab administration”: it would indicate four and 11 cycles for patients 5 and 6, respectively, as pembrolizumab was administered every 3 weeks, according to authors' note.*

‡ *No exact number of cycles has been provided by the authors.*

§ *A fifth case of a patient undergoing ipilimumab and pembrolizumab who developed an immune-related neuropathy is mentioned, but not described in the paper.*

*Ab, antibodies; A-C dissociation, albuminocytologic dissociation; GBS, Guillain-Barré syndrome; IFN-alpha, interferon-alpha; IL2, interleukin 2; IVIg, intravenous immunoglobulins; NA, not applicable; PEX, plasma exchange*.

## Discussion

Pembrolizumab-induced acute neuropathy is a rare n-irAE. It is not clear whether the association with other checkpoint inhibitors could drive synergically the onset of the condition. Based on our review of literature, neurological symptoms, such as limb weakness and/or sensory disorders, as well as brainstem and cranial nerve deficits, usually appear soon after the initiation of pembrolizumab and should be carefully investigated in order to rule out differential diagnoses, especially CNS metastases or paraneoplastic syndromes. MRI of the brain and the spine, CSF analysis, neurophysiological studies, and laboratory tests for autoimmune neuropathies and paraneoplastic syndromes are the most useful procedures for the diagnosis. For instance, in cases of polyradiculopathy, MRI of the spine with gadolinium may reveal root enhancement, although this is not a regular finding: in a recent series (16), only two of five cases with facial neuropathies and four of six cases with polyradiculoneuropathy demonstrated gadolinium enhancement of cranial nerves or spinal nerve roots, respectively. CSF may be normal in a minority of cases, whereas it usually harbors some abnormalities, such as pleocytosis with or without increased protein level (19) or albuminocytologic dissociation; the prevalence and clinical meaning of autoimmune antibodies, which may be common in n-irAE affecting the CNS [as recently reported by Sechi et al. (20)], are not clearly determined so far; finally, although data provided by different authors are heterogeneous, NCS and EMG seem to have a higher sensitivity and specificity than laboratory tests.

We described a peculiar case of a lung adenocarcinoma patient who developed an acute neuropathy of cranial nerves from pembrolizumab. Cranial nerve disorders may be observed among patients developing neuropathies from checkpoint inhibitors, as reported by Dubey et al. (16): in this series, seven out of 19 patients with peripheral n-irAEs showed cranial nerve involvement, with or without meningitis, and six had non-length-dependent polyradiculopathies with or without cranial nerve disorders. However, as far as we know, only five cases of pembrolizumab-induced isolated neuropathy of cranial nerves have been described so far: all those cases are described in melanoma patients (8, 18), while only three patients with lung adenocarcinoma have been reported, and none of them presented an exclusive involvement of cranial nerves as in our case. Whether the over-representation of melanoma patients might just reflect the larger employment of pembrolizumab in this tumor or there is a causative correlation should be investigated in further studies. Our patient shares some features with other cases reported in literature: he presented with immune-related neuropathy only after three cycles of pembrolizumab; dismissal of the drug produced clinical benefits; and antibodies of autoimmune neuropathies were not detected by laboratory tests. However, he also presented peculiar features. First, CSF analysis did not show albuminocytologic dissociation or slight pleocytosis, as commonly seen in similar cases; second, he developed a multineuropathy of cranial nerves with no involvement of the extremities; furthermore, he presented with a complex disorder of multiple nerves: in fact, symptoms and signs due to the involvement of the III, IV, VI, VII, IX, and X nerves were all present, and NCS and altered blink reflex confirmed the damage of facial nerves and revealed a subclinical impairment of the trigeminal nerves. Conversely, in other cases of isolated pembrolizumab-derived cranial nerve disorders, patients have been usually reported to have mononeuropathies (mostly facial palsies) or involvement of few cranial nerves (18). Finally, IVIg therapy (not associated to steroids) dramatically impacted the clinical course of the disease, with an improvement of both symptoms and neurophysiological tests since the first cycle: in literature, only two patients were treated with IVIg alone (9, 10), while the most common strategies were a combination of steroids, plasma exchange, and/or IVIg.

## Conclusion

In case of immune-mediated neuropathy, pembrolizumab should be dismissed immediately. According to our experience, IVIg can be a useful and effective treatment: nevertheless, a combination of steroids and/or plasma exchange should be considered based on clinical severity.

## Data Availability Statement

The original contributions presented in the study are included in the article/Supplementary Material, further inquiries can be directed to the corresponding author/s.

## Ethics Statement

Written informed consent was obtained from the patient for the publication of any potentially identifiable images or data included in this article.

## Author Contributions

All authors listed have made a substantial, direct and intellectual contribution to the work, and approved it for publication.

## Conflict of Interest

The authors declare that the research was conducted in the absence of any commercial or financial relationships that could be construed as a potential conflict of interest.
