# Supplementary material for: Pembrolizumab-Induced Isolated Cranial Neuropathy: A Rare Case Report and Review of Literature
Source: Front Neurol. 2021 May 11;12:669493. doi: 10.3389/fneur.2021.669493 (PMC8144636; doi:10.3389/fneur.2021.669493)
Supplement: Supplementary file 1 [file Data_Sheet_1.PDF]

CARE Checklist of information to include when writing a case report

CARE case report guidelines

(cc) BY-NC-ND

Topic	Item	Checklist item description	Reported on Line
Title	1	The diagnosis or intervention of primary focus followed by the words "case report"	1-2
Key Words	2	2 to 5 key words that identify diagnoses or interventions in this case report, including "case report"	26-27
Abstract (no references)	3a	Introduction: What is unique about this case and what does it add to the scientific literature?	8-10
	3b	Main symptoms and/or important clinical findings	13-16
	3c	The main diagnoses, therapeutic interventions, and outcomes	16-19
	3d	Conclusion—What is the main "take-away" lesson(s) from this case?	22-23
Introduction	4	One or two paragraphs summarizing why this case is unique (**may include references**)	66-75
Patient Information	5a	De-identified patient specific information.	78
	5b	Primary concerns and symptoms of the patient.	78-80
	5c	Medical, family, and psycho-social history including relevant genetic information	NOT APPLICABLE
	5d	Relevant past interventions with outcomes	78-80
Clinical Findings	6	Describe significant physical examination (PE) and important clinical findings.	80-81
Timeline	7	Historical and current information from this episode of care organized as a timeline	80-96
Diagnostic Assessment	8a	Diagnostic testing (such as PE, laboratory testing, imaging, surveys).	81-91
	8b	Diagnostic challenges (such as access to testing, financial, or cultural)	NOT APPLICABLE
	8c	Diagnosis (including other diagnoses considered)	82; 92
	8d	Prognosis (such as staging in oncology) where applicable	95-96
Therapeutic Intervention	9a	Types of therapeutic intervention (such as pharmacologic, surgical, preventive, self-care)	92-93
	9b	Administration of therapeutic intervention (such as dosage, strength, duration)	92-93
	9c	Changes in therapeutic intervention (with rationale)	NOT APPLICABLE
Follow-up and Outcomes	10a	Clinician and patient-assessed outcomes (if available)	93-96
	10b	Important follow-up diagnostic and other test results	94-96
	10c	Intervention adherence and tolerability (How was this assessed?)	93-94
	10d	Adverse and unanticipated events	93-97
Discussion	11a	A scientific discussion of the strengths AND limitations associated with this case report	120-133
	11b	Discussion of the relevant medical literature **with references**.	98-109
	11c	The scientific rationale for any conclusions (including assessment of possible causes)	98-133
	11d	The primary "take-away" lessons of this case report (without references) in a one paragraph conclusion	136-138
Patient Perspective	12	The patient should share their perspective in one to two paragraphs on the treatment(s) they received	NOT APPLICABLE
Informed Consent	13	Did the patient give informed consent? Please provide if requested	Yes ☒ No ☐
